# Heritable gene expression differences between apomictic clone members in *Taraxacum officinale*: Insights into early stages of evolutionary divergence in asexual plants

**DOI:** 10.1186/s12864-016-2524-6

**Published:** 2016-03-08

**Authors:** Julie Ferreira de Carvalho, Carla Oplaat, Nikolaos Pappas, Martijn Derks, Dick de Ridder, Koen J. F. Verhoeven

**Affiliations:** Department of Terrestrial Ecology, Netherlands Institute of Ecology (NIOO-KNAW), Droevendaalsesteeg 10, 6708 PB Wageningen, The Netherlands; Bioinformatics Group, Wageningen University, Droevendaalsesteeg 1, 6708 PB Wageningen, The Netherlands

**Keywords:** Apomixis, Dandelion, Functional divergence, RNA-Seq, Taraxacum officinale, Transposable elements

## Abstract

**Background:**

Asexual reproduction has the potential to enhance deleterious mutation accumulation and to constrain adaptive evolution. One source of mutations that can be especially relevant in recent asexuals is activity of transposable elements (TEs), which may have experienced selection for high transposition rates in sexual ancestor populations. Predictions of genomic divergence under asexual reproduction therefore likely include a large contribution of transposable elements but limited adaptive divergence. For plants empirical insight into genome divergence under asexual reproduction remains limited. Here, we characterize expression divergence between clone members of a single apomictic lineage of the common dandelion (*Taraxacum officinale*) to contribute to our knowledge of genome evolution under asexuality.

**Results:**

Using RNA-Seq, we show that about one third of heritable divergence within the apomictic lineage is driven by TEs and TE-related gene activity. In addition, we identify non-random transcriptional differences in pathways related to acyl-lipid and abscisic acid metabolisms which might reflect functional divergence within the apomictic lineage. We analyze SNPs in the transcriptome to assess genetic divergence between the apomictic clone members and reveal that heritable expression differences between the accessions are not explained simply by genome-wide genetic divergence.

**Conclusion:**

The present study depicts a first effort towards a more complete understanding of apomictic plant genome evolution. We identify abundant TE activity and ecologically relevant functional genes and pathways affecting heritable within-lineage expression divergence. These findings offer valuable resources for future work looking at epigenetic silencing and *Cis*-regulation of gene expression with particular emphasis on the effects of TE activity on asexual species’ genome.

**Electronic supplementary material:**

The online version of this article (doi:10.1186/s12864-016-2524-6) contains supplementary material, which is available to authorized users.

## Background

Natural plant populations reproduce mainly through sexuality. However, sex does not always increase fitness [1]. Consequently, during Angiosperm evolution, non-sexual ways of reproduction have evolved independently and recurrently [2]. Asexuality offers the ecological advantages of uniparental reproduction and enables the transmission of well-adapted gene combinations [3, 4]. It can be a less costly alternative to sexuality in sparsely inhabited, homogenous environments where abiotic factors dominate the selection regime [1, 5]. Sexual and asexual reproductions have different consequences for genome evolution, and much effort is spent on unravelling the evolutionary forces that shape genomes under the different modes of reproduction [6, 7].

Apomixis is an asexual mode of reproduction where the maternal parent produces clonal seeds [8]. It has been proposed to be induced by novel mutations and/or hybridization leading to deregulation of original sexual timing of developmental pathways [9, 10]. Polyploidy is often associated with the formation of apomict species [11] with some exceptions [12]. Consequences of hybridization and polyploidy on plant genomes and transcriptomes are diverse and cannot be entirely disentangle from the effects of asexual transition in polyploid apomicts. Chromosomal rearrangements, unequal rate of sequence evolution in duplicated copies, changes in DNA methylation and gene expression may compromise functional genome integrity [12-14]. On the other hand, the advantages of having several sets of chromosomes include buffering against deleterious mutations [15] and the creation of novel phenotypes which may promote niche specialization [16, 17]. Empirical evidence suggests that polyploid apomixis is associated with mutation accumulation and altered selection rates, as well as genome rearrangements (duplication events) and accumulation of transposable elements [18–20]. These mechanisms hint at potential rapid divergence within one asexual lineage creating appreciable heterozygosity for selection to act upon.

Under asexual genome evolution, transposable element (TE) activity and epigenetic modifications can be important sources of variation in gene regulation and are relevant for adaptive divergence to new environments [21]. For instance, TE and epigenetic molecular processes (methylation, histone modification, small RNAs) can generate heritable genetic variation in response to environmental and biotic stresses [22]. These mechanisms often act in synergy: TEs inserted near functional genes can affect their expression and act as enhancers, promoters or new regulatory elements at the genetic level [23]. Because transposable elements can escape their genomic background through recombination in sexual populations, TEs can be selected for increased transposition rates in sexual populations despite deleterious effects on their host’s fitness [24]. Conversely, asexual lineages that derived recently from sexual ancestors may inherit a subset of active TEs. The TE load can accumulate within the lineage due to less efficient purging under asexual reproduction and lack of recombination [25].

While the potential for TE activity to affect heritable variation and adaptation is evident, it remains largely unknown to what extent it is responsible for rapid divergence within asexual lineages. Here, we characterize heritable gene expression differences between accessions from a single apomictic plant lineage. The goal is to shed light on the processes involved in early divergence under asexual plant evolution, including the importance of TEs. Apomictic genotypes of the common dandelion (*Taraxacum officinale*) represent a good model system to study early divergence within asexual lineages. Derived from sexual diploid ancestors in south-central Europe [26, 27], dandelions from northern Europe are usually triploid apomicts (3x = 24). Backcrosses between polyploid (apomictic) pollen donors and sexual mother plants occur in the hybridization zone, leading to the creation of new apomictic lineages that can spread further North [26]. This process leads to high clonal diversity within the apomictic species. Distribution patterns of apomicts in the northern range have been established since the last ice age and some of these lineages have become geographically widespread [27]. Previous studies on asexual dandelions have shown epigenetic responses to abiotic and biotic stresses and demonstrated that these alterations in methylation pattern can be inherited by offspring [28, 29]. Stress responses can have an impact on transposable element activation, while methylation regulates their proliferation in the host genome.

In this paper, we use high-throughput sequencing to identify heritable transcriptomic differences between natural accessions of a single apomictic *Taraxacum officinale* lineage. Specifically, we address the following questions: (1) To which extent does expression divergence occur in a clonal lineage and how important is TE involvement in this divergence? (2) Is there functional divergence between the geographically separated clone members? Here, we provide evidence for heritable gene expression divergence occurring within one single clonal lineage with differential TE activity among apomicts. This study also suggests that some functionally relevant genes and pathways are affected during recent asexual evolution.

## Methods

### Plant material and sampling

Natural accessions of the *Taraxacum officinale* apomictic genotype Macranthoides were sampled in Germany and the Czech Republic during spring 2012. Macranthoides is a common and geographically widespread apomictic dandelion microspecies that can be identified phenotypically by expert taxonomists [30]. Based on microsatellite genotyping, Macranthoides (and several other dandelion apomict microspecies) are thought to be of monoclonal origin (tracing back to a single apomictic founding event) as opposed to a multiclonal origin (assemblages of multiple, genetically related lineages that trace back to independent founding events) [30]. Indeed, Kirschner and co-workers showed that within-microspecies genetic analysis typically reveals incidental deviations from a single common multiloci genotype which is fully consistent with unique founding event followed by mutation accumulation under asexual reproduction.

**Fig. 1:**
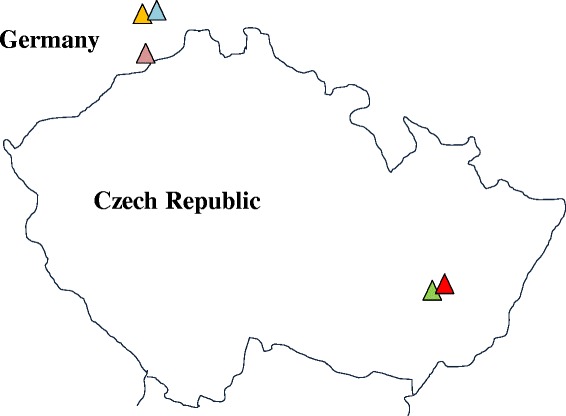
Sampling sites of the five accessions in the Czech Republic and Germany. Accessions 12 and 13; and 11 and 8, were collected from the same field

Accessions were chosen to represent different geographical scales: five accessions were sampled on three different fields hundreds of kilometers apart (Fig. 1). Apomictic clonal seeds were propagated for one generation in a common greenhouse environment. For all experiments, seeds were surface-sterilized with a 0.5 % Sodium Hypochlorite solution and were germinated on agar plate (0.8 %) in petri dishes for 10 days in a climate chamber (10 h dark/14 h light, 15/20 °C). Then, seedlings were transplanted to individual pots (80 % potting soil with 20 % pumice) and grown in the greenhouse until leaf and/or seed collection. DNA from first-generation offspring plants was extracted from young leaves using a CTAB-based protocol [31, 32] to validate their common and unique genotype origin using microsatellite markers. Samples were screened at eight microsatellites markers developed for *T. officinale* using an ABI PRISM genetic analyzer [33, 34]. Five accessions with identical multi-locus genotypes were chosen for subsequent transcriptomic experiments (Additional file 1); the observed pattern of identical allele polymorphisms between accessions confirms the uniclonal nature of Macranthoides [30] and is consistent with strict apomictic reproduction.

Apomictic dandelions produce meiotically unreduced embryo sacs derived from unreduced megaspores, which are formed by a first meiotic division restitution [35]. The second meiotic division produces two megaspores (instead of four). The surviving unreduced megaspore develops parthenogenetically to create a new embryo thought to be genetically identical to the mother plant [36].

After greenhouse propagation for one generation, for each of the five accessions 70 propagated seeds were chosen from one seed head, weighed (in pools of ten seeds) and germinated in a climate chamber as described above. Seedlings were transplanted to pots and grown under greenhouse conditions for one week. Plants were then divided into two groups: one group was grown under greenhouse conditions until tissue sampling; the second was transplanted into a mowed and manually tilled field plot (near Wageningen, NL) in a pasture with an established natural *T. officinale* population (further referred to as semi-natural field).

For both experiments we followed the same randomized block experimental design with five accessions and eight blocks, where each block contained four replicate plants per accession. Blocks were ordered fully randomized (160 plants in total per experiment). For subsequent RNA extraction and transcriptome sequencing we pooled eight individuals to form one biological replicate (one individual per block), resulting in four independent pools per accession. After three and seven weeks of growth (greenhouse and semi-natural field respectively), for each individual two discs from one young fresh leaf were punched. Leaf tissue discs from eight individuals were pooled in one tube, directly frozen in liquid nitrogen and stored at −80 °C until RNA extraction. In total, this yielded 20 tubes per experiment, four each for the five accessions.

### RNA extraction and DNase treatment

Total RNA was isolated using ground leaf tissue and adding TRIzol reagent (Invitrogen) according to manufacturer protocol with minor modifications as follows. Briefly, after transferring aqueous phase to a new tube, RNA was precipitated twice using isopropanol and sodium acetate (3 M, pH 5.2); pellet was then washed and re-suspended in 50 μl of DNase/RNase-free water. Quality was checked on agarose gel electrophoresis and concentration on a NanoDrop 1000 spectrophotometer. For each sample, 10 μg of total RNA was then treated with TURBO DNA-free kit (Ambion AM1907). Integrity and concentration were again checked using agarose gel electrophoresis and spectrophotometer.

### External RNA control consortium spike-in control, library preparation and Illumina sequencing

For each experiment, 20 RNA libraries were prepared from 1 μg of total RNA using the Illumina TruSeq RNA Sample prep Kit v2. Before proceeding with the manufacturer’s instructions, we added in-library RNA controls to verify accuracy of the gene expression quantification for each individual library [37]. Ninety-two synthetic External RNA Control Consortium (ERCC) RNA spike-in control sequences [38] were added to each *T. officinale* total RNA sample (4 μl of Mix 1, diluted to 1/400 from Ambion, Life Technologies Inc.). The pool of transcripts was designed to closely represent eukaryotic mRNAs with balanced GC content and length from 250 to 2000 nucleotides [37]. For counting reads matching each of the 92 synthetic sequences, a fasta file containing all sequences was downloaded from the website of Ambion (Life Technologies Inc.) and used as a reference to map the reads sequenced as described later in this section.

For each experiment, all 20 libraries were indexed and checked on an Agilent 2100 Bioanalyzer. Real-time PCR was also performed using the KAPA SYBR® FAST Universal qPCR Kit (KAPA Biosystems) to correctly quantify concentrations for each library and pool them accurately in equimolarity. Per experiment all 20 libraries were multiplexed into a single pool and 10 nM of both pools were sequenced on four lanes (two lanes per experiment) using an Illumina HiSeq 2000 platform to generate 100 bp paired-end sequences.

### Assembly of transcripts

As no reference transcriptome is currently available for *T. officinale*, a *de novo* reference was assembled using all 40 libraries sequenced. Raw reads were first trimmed for adaptors and low quality reads (Phred score < 33) using the fastq-mcf tool from ea-utils [39]. Additionally, the first 10 nucleotides of all reads (both reverse and forward) were trimmed using seqtk (https://github.com/lh3/seqtk). This is known to improve assembly of full length *de novo* transcripts [40]. Assembly was performed using Trinity version r20140413 [41], using default parameters with a minimum kmer coverage of two to get rid of possible sequencing errors.

### Annotations and gene ontology

Transcripts were annotated using BLAST, KEGG and SwissProt protein (http://web.expasy.org/) databases (BLASTx, *E* < 1e-05), as well as the *Arabidopsis thaliana* Protein Coding Sequence database (BLASTn, *E* < 1e-05). Output files were parsed to retain the best blast hit (best *E*-value) for each transcript with more than one hit. The Blast2Go suite was then used to generate gene ontology terms based on the BLAST outputs [42]. Strict parameters were used allowing a BLAST high-scoring segment pair length of 33 and a minimum coverage of the query of 33. Gene ontology enrichment analyses (Fisher’s test on the most specific ontologies, adjusted *p*-value < 1e-05, following recommendations from [43]) were also conducted using the Blast2Go suite for the set of differentially expressed genes (DEGs) against a background of all *de novo* assembled genes.

### Mapping and identification of differentially expressed genes

Reads from each library were mapped against the new reference transcriptome using Bowtie [44] with default parameters. The software RSEM [45] was also used to count the number of reads matching each putative gene.

To perform the differential expression analysis, the Bioconductor DESeq package was used on raw count numbers [46]. Both experiments were analyzed separately giving more confidence into overlapping DEGs. Filtering was performed over all counts, removing the lowest 40th percentile of transcripts based on mapped read counts [47]. Using DESeq, samples were normalized and differential expression between accessions was tested for each transcript separately using a statistical model based on the negative binomial distribution (see [46]). More precisely, two models are specified: the full model regresses gene expression on accessions while the reduced model tests for the null hypothesis. For each gene, the generalized linear model is fitted according to the two models which are then compare to infer whether accessions improves the fit and hence has significant effect. Transcripts showing a significant accession effect after correcting for multiple testing at a FDR threshold of 0.05 [48] were considered as differentially expressed. Principal Coordinate Analyses (PCoA) were performed using GenAlEx [49], based on pairwise Euclidean distances as calculated by DESeq from normalized read counts. Additionally, broad-sense heritability (H^2^) was estimated for all genes as the variance component due to the accession effect divided by the total variance (accessions plus residual variance components). Variance components were estimated using PROC MIXED in SAS 9.2.

### Gene network analysis

To identify and visualize networks and highly connected genes (which interact indirectly through RNA and protein expression products with many other genes) among the list of DEGs, Virtual Plant 1.3 was used (www.virtualplant.org). We were able to detect and quantify known molecular interactions in the different sets of DEGs previously found from public databases using *A. thaliana* annotations (see [50] for details on databases). More specifically, the tool “network statistics” was used to generate tables of the most highly connected nodes in the network and to pinpoint ecologically relevant genes and functions. Functional annotations of all highly connected genes were then retrieved from the TAIR website (www.arabidopsis.com).

### SNP analysis

To estimate genetic divergence between the five accessions, SNPs were called from the RNA-Seq data in a two-step process. First, a set of high-confidence SNPs was identified within each accession separately. Second, these sets were evaluated in all five accessions to distinguish SNPs that are shared between all accessions (which reflect within-individual allelic polymorphisms that predate the most recent common ancestor of the accessions within the apomictic lineage) from SNPs that differentiate between accessions.

To improve nucleotide coverage at all loci, trimmed and quality-filtered reads were concatenated per accession, pooling together biological replicates and both experiments. Using the BWA-backtrack program and default value parameters, resulting sequences were mapped to the Trinity reference transcriptome assembly [51]. Mapping statistics were calculated with the FlagStat utility from SAM tools [52]. Alignments were further processed using Picard tools (http://broadinstitute.github.io/picard) to add read group information for each accession, tag duplicates and to sort and index the final BAM files.

The Freebayes analysis pipeline was used for SNP calling (Garrison and Marth, unpublished data) with the ploidy parameter set to three. Quality was filtered using the vcflib tool (downloaded from https://github.com/ekg/vcflib). Read coverage for each SNP position in all accessions was checked with Bedtools [53]. Due to the large amount of SNPs in the transcriptome, reflecting high intra-individual heterozygosity, we applied strict filters (Quality >200 and Read coverage >50 in each accession) to obtain a finalized set of high-confidence SNPs that are not shared between all five accessions. This subset of high-confidence SNPs should accurately capture divergence of the accessions under asexual reproduction within the apomictic lineage. Using custom R scripts [54], a binary SNP matrix was built and processed to calculate pairwise Hamming distances between accessions [55] using the package APE [56]. The hclust function generated the hierarchical clustering tree (from the fastcluster package [57]). Finally, in order to assess correlations between geographic, genomic and transcriptomic distances, Mantel tests were performed on the respective distance matrices using ZT software [58].

## Results

### RNA-Seq data analysis, transcriptome assembly and annotation

Libraries of the greenhouse experiment yielded a total amount of 288.52 million raw 100 bp paired-end reads, of which 181.20 million high-quality reads were used for *de novo* assembly. Libraries of the semi-natural field experiment generated 419.45 million raw reads of which 183.9 million high-quality reads were retrieved. The construction of the assembly used all 40 libraries in order to build a *de novo* transcriptome as complete as possible. Trinity assembled 123,232 transcripts belonging to 77,433 clusters representing putative genes. Transcript lengths ranged between 500–14,514 bp, with an average and median of 908 bp and 584 bp respectively, and N50 of 1451 bp. This *de novo* transcriptome represents a total sequence of 111.95 Mb.

Assembled transcripts were first annotated using protein databases (SwissProt and KEGG) and then using a nucleotide database of coding sequences from *A. thaliana*. Out of the 123,232 transcripts, 42,592 showed significant matches to SwissProt, 8,476 to KEGG and 39,685 to *A. thaliana* coding sequence database. Approximately 35 % of unique consensus sequences were successfully annotated in this study. This ratio is relatively low compared to other *de novo* transcriptome sequencing studies for non-model species due to short length of assembled transcripts. Lacking a genomic reference in addition with low-coverage RNA-Seq limits our ability to build full-length transcripts.

For all libraries, ERCC spike-in reads were retrieved and mapped back to their known sequence reference. Approximately 30,000 reads (range ±12,000) were mapped for each library, representing less than 0.3 % of total reads. Out of the 40 libraries, two produced low ERCC spike-in sequence counts (two replicates of accessions 3 and 12). However, transcript expression levels as well as KAPA quantification of these two libraries were consistent with others; thus, low ERCC spike counts were presumed due to mis-pipetting of the ERCC spikes and these libraries were retained in the analysis.

Due to size selection during library preparation, ERCC sequences less than 300 bp as well as sequences longer than 1,990 bp were under-represented. Nevertheless, spike-in concentrations and observed read numbers were highly correlated for all libraries. For each experiment four sub-groups of spikes (with different concentrations and sequence size, as recommended by the manufacturer) were used, and for all subgroups and libraries the correlations between input concentration and read output ranged from *R*^*2*^ = 0.9522 to *R*^*2*^ = 0.9951 (Additional file 2). The use of ERCC is then a good alternative to quantitative real time PCR to assess sensitivity and accuracy of our transcriptomic libraries, supporting quantitative interpretation of the read output.

### Differential transcript abundance between accessions

Reads were mapped back to the reference transcriptome and counted. Differentially Expressed Genes were detected when transcript levels of expression were significantly different in at least one accession. Out of the 77,433 putative genes assembled, 45,939 (semi-natural field) and 45,814 (greenhouse) passed the overall count filtering for differential gene expression analysis. The analysis revealed evidence for 504 DEGs between accessions in the semi-natural field environment and 369 in the controlled environment (Additional files 3 and 4 respectively). There was strong overlap in DEGs between the two test environments: 295 transcripts were differently expressed between accessions in both experiments, with 209 only differentially expressed in the semi-natural field experiment and 74 only in the controlled experiment. Differentially Expressed Genes exhibit high heritability indexes (0.733 ± 0.187 and 0.737 ± 0.175 for the field and greenhouse experiments, respectively) compared to overall genes (0.065 ± 0.128 and 0.056 ± 0.119 for the field and greenhouse experiments, respectively) (Additional file 5).

**Fig. 2:**
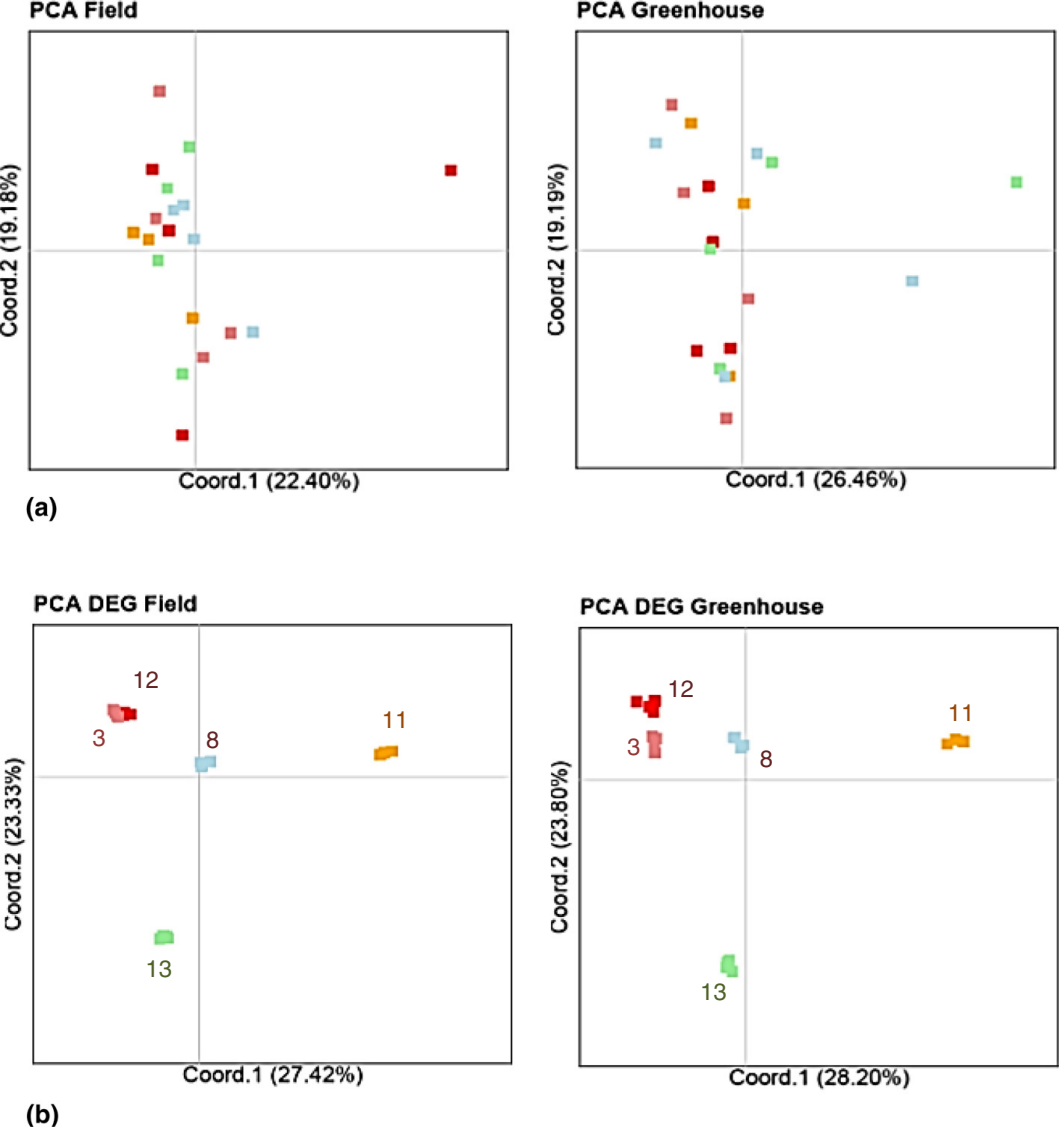
Principal Coordinate Analyses of gene expression patterns, based on pairwise Euclidean distances as calculated by DESeq from normalized read counts of the five accessions on (**a**) all transcripts and (**b**) only genes differentially expressed (DEG) for each experiment (see Fig. 1 for sampling locations)

Principal Coordinate Analyses (PCoA) of expression levels based on all analyzed putative genes showed no clear pattern over all samples and biological replicates (Fig. 2a), confirming overall transcriptomic uniformity of the accessions. In contrast, PCoA of expression levels based only on the subset of DEGs exhibited a characteristic pattern that was similar for both experiments (Fig. 2b): biological replicates clustered together while all five accessions were clearly separated. Opposite to what was expected, accessions collected from the same field did not show the most similar transcriptomic profiles (Figs. 1 and 2b).

### Gene ontology and functional enrichment of DEG

In DEG lists, 36.5 % and 35.5 % found a blast hit against *A. thaliana* and the SwissProt Database, respectively for the field and greenhouse experiments (Additional files 3 and 4). A large number of DEGs with descriptive functional annotations were retrieved as protein domains of transposable elements (34.3 % of the annotated transcripts, Additional file 6). Most of these assembled transcripts were too short to have the full transposable element sequence. However, we detected protein domains of some Long Terminal Repeat retrotransposons (Class I transposable elements *Copia* and *Gypsy*) represented by GAG and POL genes (with Protease, Reverse Transcriptase, RNase H, Integrase and Endonuclease domains). Non-LTR retrotransposons were also well-represented with ORF2 and Reverse Transcriptase domains from putative *LINE-*1 and *Tnt*-1 elements.

Functional annotation using Blast2Go and *A. thaliana* as a reference, retrieved annotations for 23,242 out of the 77,433 putative genes, resulting in 8,430 different GO categories. For each experimental condition, we compared gene ontology of DEGs to all genes that found a homology hit against *A. thaliana*. Differentially Expressed Genes from the greenhouse treatment were specifically enriched for molecular functional categories such as “RNA-directed DNA polymerase activity”, “DNA binding”, “aspartic-type endopeptidase” and “endonuclease activity” as well as “zinc-ion binding” (Table 1). Biological process categories enriched in the DEG list included “DNA integration”, and cellular components included “central vacuole” (Table 1). Similarly, in the semi-natural field experiment, DEGs were enriched for molecular function categories “RNA-directed DNA polymerase” and “aspartic-type endopeptidase” activities as well as “zinc-ion binding”. Two molecular functions are specific to this treatment: “Ribonuclease activity” and “RNA binding”; and one term for biological process, “DNA recombination” (Table 2). These GO categories (specifically “RNA-directed DNA polymerase”, “aspartic-type endopeptidase activities”, “ribonuclease activity” and “RNA binding”) reflect activity of transposable elements with an over-representation of Gag/Pol and Transposase domains [59]. Zinc-ion binding function indicates a role for transcription factors, *i.e.* promoters or repressors in the recruitment of RNA polymerase controlling rate of transcription [60]. Biological processes (specifically DNA binding, integration and recombination) are involved in the maintenance and integrity of the DNA and potentially counteract the effects of repeat proliferation in the genome.

**Table 1:** Specific GO-enrichment analysis in greenhouse experiment

GO-ID	Term	Category	FDR	*P*-value
GO:0003964	RNA-directed DNA polymerase activity	F	1.73E-12	3.46E-16
GO:0003676	nucleic acid binding	F	3.12E-10	1.40E-13
GO:0004190	aspartic-type endopeptidase activity	F	3.36E-07	2.49E-10
GO:0015074	DNA integration	P	1.11E-06	9.99E-10
GO:0004519	endonuclease activity	F	3.28E-06	3.28E-09
GO:0008270	zinc ion binding	F	4.15E-05	6.24E-08
GO:0042807	central vacuole	C	2.85E-02	6.28E-05

Categories: P: biological process; F: molecular function; C: cellular component

**Table 2:** Specific GO-enrichment analysis in field experiment

GO-ID	Term	Category	FDR	*P*-value
GO:0003964	RNA-directed DNA polymerase activity	F	1.42E-20	1.22E-24
GO:0004190	aspartic-type endopeptidase activity	F	1.60E-07	1.10E-10
GO:0015074	DNA integration	P	1.89E-07	1.47E-10
GO:0004523	ribonuclease H activity	F	4.74E-07	4.09E-10
GO:0008270	zinc ion binding	F	5.80E-04	1.15E-06
GO:0003723	RNA binding	F	1.82E-03	4.23E-06
GO:0006310	DNA recombination	P	8.91E-03	2.23E-05

Categories: P: biological process; F: molecular function; C: cellular component

### Gene network analysis

To zoom in on relevant genes and pathways affected during divergence between the five apomictic lineage members, a network analysis was performed on all annotated DEGs. This analysis enables to expose highly connected DEG transcripts using annotations and known molecular interactions from *A. thaliana*. We present and discuss genes and associated functions with more than 10 connections, in both environments separately.

In the greenhouse experiment, 67 DEGs had a blast hit against *A. thaliana* genes and 15 of which were found as highly connected genes (Additional file 7). Three genes showed more than 10 connections with other DEGs. The first one encodes for the *Beta-ketoacyl synthase* (At1g74960) and is involved in fatty acid elongation [61]. The second (*Peanut1*, At5g22130) is part of chemical reactions and pathways resulting in the formation of cell membranes [62]. Lastly, *Zeaxanthin Epoxidase* (At5g67030) is part of the abscisic acid biosynthetic process.

DEGs from the semi-natural field experiment included 27 highly connected genes (out of 95 transcripts with a blast hit against *A. thaliana*) (Additional file 8). Four genes showed more than 10 connections with other DEGs. Three of these (*Delta 9 Desaturase 1*, *Fatty Acid Biosynthesis 1* and a putative gene encoding a peroxisomal protein, At1g06080, At1g74960, and At4g05160 respectively) are involved as previously described, in elongation of fatty acids and their activation through esterification, all three are involved in jasmonic acid biosynthesis [61]. The gene showing the most connections (*Delta 9 Desaturase 1*) is known to produce long fatty-acid chains and is part of a defense mechanism in response to insects [61]. The last gene (*Squalene Epoxidase 1*, At1g58440) has been speculated to have squalene monooxygenase activity and to be involved in the formation of terpenoids, polysaccharides and sterols [63].

### Variant calling and genetic clustering analysis

**Fig. 3:**
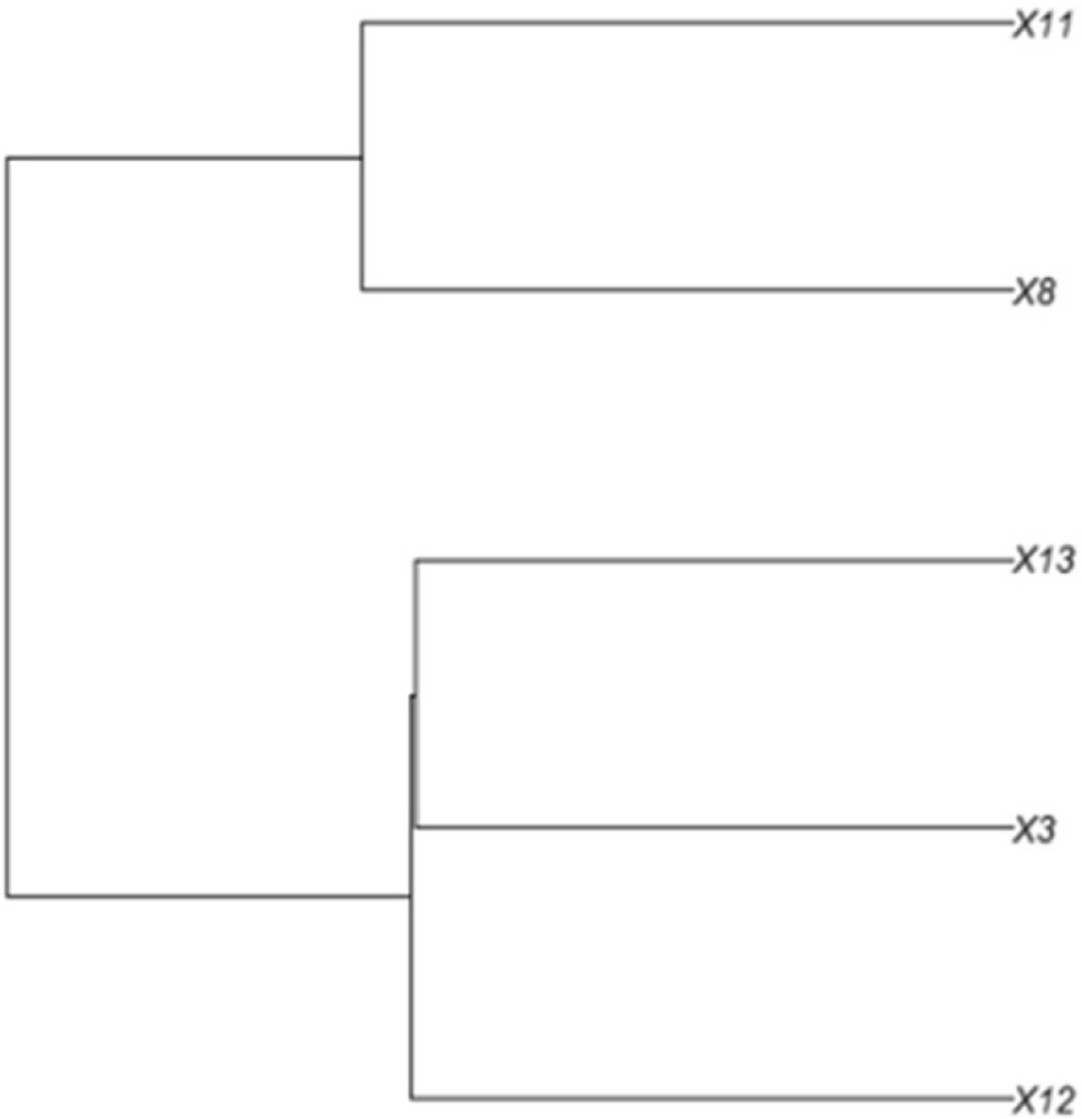
Genetic clustering based on a subset of high-confidence SNPs. Pairwise distances were calculated based on Hamming distance and visualized using hierarchical clustering

More than 90 % of the input reads were mapped back to the reference transcriptome, the majority of these (more than 83 %) properly paired across all accessions (Additional file 9). A total of 302,969 SNPs were called that passed the quality threshold of Q200 (Phred score). After discarding SNPs with less than 50 read coverage in at least one accession and SNPs that reflected allelic variation shared between all five accession, a high-confidence subset of 4,091 SNPs that discriminated between the accessions were kept for subsequent analyses. Pairwise SNP distances are presented in Additional file 10. Genetic clustering analysis based on a hierarchical clustering tree reveals two distinct clusters: one comprised by accessions 12, 3 and 13 and another comprised by accessions 8 and 11 (Fig. 3, Additional file 10).

Mantel correlations were performed between geographic distances, overall genetic distances and expression distances, and expression distances based only on DEGs of both field and greenhouse experiments (Additional file 11). Consistent with the hierarchical clustering based on SNPs (Fig. 3), geographic and genetic distances correlated only weakly (*r* = 0.18, *p* = 0.13); this is due to a German accession clustering genetically with the accessions from eastern Czech Republic (accession 3, see Fig. 3). Furthermore, observed expression differences between the accessions were only weakly correlated to genomic divergence (correlation between SNP distances and expression distances based on all transcripts: *r* = 0.04-0.18, *p* = 0.35-0.28 respectively for field and greenhouse experiments and between SNP distances and DEG-based expression distances: *r* = 0.29-0.13, *p* = 0.18-0.31 respectively for field and greenhouse experiments). The absence of a strong correlation between expression and genomic divergence is also visible by comparing Figs. 2 and 3: the two SNP-based clusters (8 and 11 versus 3, 12 and 13; Fig. 3) are not obviously similar to the expression-based clusters (Fig. 2b).

## Discussion

This study aimed to shed light on early evolutionary divergence under asexual reproduction by analyzing heritable gene expression differences between apomictic clone members of a single widespread common dandelion lineage. Among the set of DEGs a high proportion of TE-associated genes were retrieved, indicating TE activity as a generator of rapid heritable differences between clone members. In addition, expression analyses identified differences between accessions for specific pathways potentially involved in abiotic and biotic stress responses, which might reflect functional divergence in response to habitat differences within this geographically widespread apomictic lineage.

### Generating heritable variation within a clonal lineage: transposable element activity

A striking result observed in our DEG set was the large number of transposable elements and protein-related sequences annotated as transcription factors and transposases. Transposable elements are known to be predominant in plant genomes [64]. Whole-genome analyses in plants reveal that the majority of transposable elements are inactive and silenced by DNA methylation and small RNAs [65]. However, studies in different plant species suggest activation of some transposable elements following environmental and/or genomic stresses (reviewed in [66, 67]). Activation of TEs can reflect genomic events that lineages have experienced in their evolutionary history such as polyploidy, hybridization, chromosomal rearrangements or loss of meiosis. Large differences in transcription and transposition of TEs can also be observed within a single plant species (in *Oryza sativa* [68] and in maize [69]), which demonstrates the dynamic nature of TEs within plant genomes.

In apomictic dandelions, our finding that TE-associated genes represent an appreciable fraction of expression divergence between apomictic clone members demonstrates that TE activity plays an important role in early evolutionary divergence in asexuals. Observed variation in TE transcription may be a by-product of both the hybridization event that gave rise to the apomictic lineage (potentially affecting methylation patterns and TE release) and apomixis [28]. Empirical evidence is still scarce to conclude whether apomixis should be associated to TE proliferation or decline [70], and this may also depend on the age of the apomictic lineage. Over longer time scales, asexual lineages with high TE abundance are supposed to go extinct while lineages with low TE load are more likely to persist [25]. However, after a recent transition from sexual to asexual reproduction, TE load in asexual lineages may increase due to the effect of inheriting a subset of actively transposing TEs in combination with less efficient purging of deleterious mutation under asexual reproduction [71], and/or due to re-activation of transposable elements after the polyploidization event that often accompanies the transition from sexual to apomictic reproduction. We assume that the divergence observed in our study mostly reflects short-term effects of the transition to apomixis from sexual ancestral genotypes. However, further genomic comparisons between sexual and asexual dandelion genotypes will be required to shed more light on this.

Differential TE activity observed among the five accessions can also be triggered by recently experienced environmental stress conditions. Biotic and abiotic environmental stresses have been demonstrated to activate TE transcription and to some extent proliferation [72–74]. One of the earliest and most studied examples is the retrotransposon *Tnt1* found in *Nicotiana tabacum* [75]. This element can be induced by bacterial and microbial factors, wounding, pathogens, jasmonic acid and salicylic acid. Since then, numerous retrotransposons were found affected by external conditions [72].

Compared to random genetic mutations, TE activation can be a source of relatively rapid functional divergence because TEs can be intimately linked to gene regulatory networks [21]. This is particularly relevant in polyploid species, where the deleterious effects of transposable element activity and transposition are buffered due to genome redundancy [11]. Transposable elements are tightly associated with DNA methylation patterns (that allow silencing and inactivation of TEs) and produce molecules such as proteins or small RNAs that can affect nearby or more distant genes. Transposable elements can therefore be a source of new gene regulatory pathways by acting as enhancer elements or promoters (*Cis* effect). Furthermore, recruiting epigenetic regulation to the insertion locus of the TE can alter expression of neighboring genes (reviewed in [23]). Lastly, some examples have been reported of TEs as a source of new genes through sequence acquisition of functional genes within TEs [76, 77]. One of the best-known examples in *Arabidopsis* revealed a high degree of sequence similarity between the *FAR1* and *FHY3* genes to MULE transposases [78] that act as transcription factors during far-red light signaling [79]. As shown in this study, effects of TEs on gene regulation may be surprisingly common: a recent study comparing *A. thaliana* and its close relative *Schrenkiella parvula*, an extremophyte adapted to multi-ion salt shores, showed that repetitive sequences are overrepresented in genes that are differentially expressed between the two species. The authors suggest that TE activity facilitated adaptive evolution to the salt shore habitat in *S. parvula* [80].

### Evidence for early functional divergence in clonal species

Network analysis pointed to expression divergence between the apomictic lineage members in pathways related to acyl-lipid metabolism and also abscisic acid (ABA) metabolism. These pathways are associated with plant growth as well as biotic and abiotic stress responses. First, within the acyl-lipid metabolism, which are the first form of carbon and energy storage in seeds, we observed highly connected transcripts playing a role in fatty acid synthesis, elongation and export. These molecules are also the major component of cutin and cuticular waxes that prevent the plant from desiccation, pathogens and other stresses in leaves [81]. Polyunsatured fatty acids such as linoleic and linolenic acids (the longest chains) are abundant in chloroplast membranes and can oxidize to produce corresponding fatty acid hydroperoxides [82]. These molecules are substrates for different classes of oxylipins including jasmonates [83] which are important in plant growth and development as well as in biotic and abiotic stress responses. The role of jasmonates in plant defense signaling in response to insect attacks is very specific through the activation of downstream genes after wounding and infestation [84]. Additionally, the ABA biosynthesis pathway through the *Zeaxanthin epoxidase* (ZEP) enzyme is associated with functional divergence. ZEP is an enzyme responsible for the biosynthesis of the first component of the ABA pathway: *Violaxanthin*, from carotenoids [85]. In *Arabidopsis*, expression of ZEP increased in response to osmotic stress and ABA treatment in both roots and shoots [86]. Identification of these specific functional pathways that are preferentially enriched in our clonal accessions is hinting at potential candidate genes and metabolisms to address functional divergence under asexuality.

### Expression divergence between apomictic clone members is not fully explained by genomic divergence

Genomic distances between the accessions reflect in part the geographic clustering of the sampling locations; however, accession 3 is genetically more similar to samples from distant sites than from the nearby site. Our data suggest two different sub-clonal lineages within the Macranthoides lineage that may both be widespread. Interestingly, a similar observation was made in another apomictic system where distant sub-lineages can be found at the same location [18].

Similarly, expression divergence and genomic divergence showed some association, but correlation was rather weak. It could be that a large fraction of the expression divergence is caused by few mutations that are not well correlated to genome-wide genetic divergence. Alternatively, other mechanisms than mutation accumulation may affect heritable transcriptomic differences between the accessions. One possibility is that epigenetic mechanisms are causing heritable expression changes between the accessions: their heritable but dynamic nature could produce expression patterns that differ stochastically between accessions or that reflect environmental conditions in previous generations rather than genomic similarity. In a similar way, maternal effects from the greenhouse seed propagation generation could affect expression patterns. An alternative explanation is that technical biases were introduced from our approach to infer genomic divergence from RNA-Seq data. Although this is a common approach [19], it is possible that allele-specific expression in some of the accessions contributed SNPs that are interpreted as divergent between the accessions while in fact they may be shared between all accessions; this potentially leads to inaccurate estimates of genomic divergence. Ultimately, further genomic and epigenetic variation analyses in the five accessions are needed to unravel mechanisms that cause early transcriptomic divergence in this species.

### Availability of data and materials

The datasets supporting the results of this article are included within the article and its Additional files. The reference transcriptome and raw read mapping tables have been submitted to the Dryad database repository under identifier number doi:10.5061/dryad.6p2n6. No field work permissions were needed for seed collecting at the stated locations.

## Conclusions

We provide insights into the mechanisms involved in the early evolutionary steps of asexual evolution in the apomictic dandelion. More specifically, we show that TE activity is a main determinant of divergence within one clonal asexual lineage and we identified candidate TEs and functional pathways affecting heritable expression divergence within the lineage. These findings will be the starting point of further genomic and epigenetic variation analyses on host silencing and *Cis*-regulation of gene expression.

## Additional files

Additional file 1: Presentation of the five accessions, microsatellite data for eight markers and sampling sites. (DOCX 15 kb) Additional file 2: Mapping results of ERCC spike-in control and correlations with theoretical concentrations. (XLSX 62 kb) Additional file 3: Differentially expressed genes identified using DESeq, heritability index and annotations from SwissProt and *Arabidopsis thaliana* coding sequence databases for the semi-natural field experiment. (XLSX 9161 kb) Additional file 4: Differentially expressed genes identified using DESeq, heritability index and annotations from SwissProt and *Arabidopsis thaliana* coding sequence databases for the greenhouse experiment. (XLSX 61 kb) Additional file 5: Distribution of heritability frequencies in all genes and subset of differentially expressed genes (DEGs) in the greenhouse (A) and semi-natural field (B) environments. (DOCX 23 kb) Additional file 6: Assembled transcripts annotated with transposable element or repeat description from the SwissProt database. (XLSX 12 kb) Additional file 7: Genes highly connected from overall differentially expressed greenhouse genes (67 genes); in grey, DEGs unique to greenhouse conditions (in white common in both experiments). Annotations and functions were retrieved from the TAIR website (www.arabidopsis.org). (XLSX 10 kb) Additional file 8: Genes highly connected from overall differentially expressed semi-natural field genes (67 genes); in grey, DEGs unique to field conditions (in white common in both experiments). Annotations and functions were retrieved from the TAIR website (www.arabidopsis.org). (XLSX 11 kb) Additional file 9: Read mapping statistics of the SNP analysis on the reference *de novo* transcriptome assembly per accession. (RTF 59 kb) Additional file 10: Pairwise SNP distances between accessions after stringent filtering steps (Quality >200 and Read coverage >50 in all accessions) using Freebayes. (DOCX 14 kb) Additional file 11: Matrix comparison of Mantel’s tests (r = coefficient of correlation, p = p-value). (DOCX 13 kb)
