# Supplementary material for: Heritable gene expression differences between apomictic clone members in Taraxacum officinale: Insights into early stages of evolutionary divergence in asexual plants
Source: BMC Genomics. 2016 Mar 8;17:203. doi: 10.1186/s12864-016-2524-6 (PMC4782324; doi:10.1186/s12864-016-2524-6)
Supplement: Additional file 1: — Presentation of the five accessions, microsatellite data for eight markers and sampling sites. (DOCX 15 kb) [file 12864_2016_2524_MOESM1_ESM.docx]

| Acc. | Sample Name | M58 | M44B | M31 | M78 | M61 | M67 | M72 | M143 | Country of origin | Coordinates |
| --- | --- | --- | --- | --- | --- | --- | --- | --- | --- | --- | --- |
| 11 | Macra11 | 104-129- | 176- - | 253-257- | 164-176- | 134-141- | 203-216-230 | 185- - | 238-248- | Germany | 50° 53' 48'' N 13° 50' 03'' E |
| 12 | Macra  1280-S1 | 104-129- | 176- - | 253-257- | 164-176- | 134-141- | 203-216-230 | 185- - | 238-248- | Czech republic | 49° 20' 56'' N 17° 18' 31'' E |
| 13 | Macra  1280-S2 | 104-129- | 176- - | 253-257- | 164-176- | 134-141- | 203-216-230 | 185- - | 238-248- | Czech republic | 49° 20' 56'' N 17° 18' 31'' E |
| 3 | Macra3 | 104-129- | 176- - | 253-257- | 164-176- | 134-141- | 203-216-230 | 185- - | 238-248- | Germany | 50° 47' 15'' N 13° 51' 22'' E |
| 8 | Macra8 | 104-129- | 176- - | 253-257- | 164-176- | 134-141- | 203-216-230 | 185- | 238-248- | Germany | 50° 53' 48'' N 13° 50' 03'' E |
