# Supplementary material for: Heritable gene expression differences between apomictic clone members in Taraxacum officinale: Insights into early stages of evolutionary divergence in asexual plants
Source: BMC Genomics. 2016 Mar 8;17:203. doi: 10.1186/s12864-016-2524-6 (PMC4782324; doi:10.1186/s12864-016-2524-6)
Supplement: Additional file 5: — Distribution of heritability frequencies in all genes and subset of differentially expressed genes (DEGs) in the greenhouse (A) and semi-natural field (B) environments. (DOCX 23 kb) [file 12864_2016_2524_MOESM5_ESM.docx]

**B.**

**A.**
