# Supplementary material for: Heritable gene expression differences between apomictic clone members in Taraxacum officinale: Insights into early stages of evolutionary divergence in asexual plants
Source: BMC Genomics. 2016 Mar 8;17:203. doi: 10.1186/s12864-016-2524-6 (PMC4782324; doi:10.1186/s12864-016-2524-6)
Supplement: Additional file 9: — Read mapping statistics of the SNP analysis on the reference de novo transcriptome assembly per accession. (RTF 59 kb) [file 12864_2016_2524_MOESM9_ESM.rtf]

	3	8	11	12	13	
Input reads	223,205,072	190,258,354	230,041,130	234,320,418	242,178,302	
Mapped
(%)	205,845,661 (92.22)	175,222,600 (92.10)	212,901,342 (92.55)	214,678,066 (91.62)	223,706,146 (92.37)	
Properly paired (%)	190,734,592 (85.45)	162,350,596 (85.33)	199,536,898 (86.74)	195,182,744 (83.30)	208,264,444 (86.00)	
% Singletons	1.99	2.03	1.96	2.03	1.94	
