# Supplementary material for: Heritable gene expression differences between apomictic clone members in Taraxacum officinale: Insights into early stages of evolutionary divergence in asexual plants
Source: BMC Genomics. 2016 Mar 8;17:203. doi: 10.1186/s12864-016-2524-6 (PMC4782324; doi:10.1186/s12864-016-2524-6)
Supplement: Additional file 10: — Pairwise SNP distances between accessions after stringent filtering steps (Quality >200 and Read coverage >50 in all accessions) using Freebayes. (DOCX 14 kb) [file 12864_2016_2524_MOESM10_ESM.docx]

|  | X3 | X8 | X11 | X12 |
| --- | --- | --- | --- | --- |
| **X8** | 2378 |  |  |  |
| **X11** | 1885 | 1597 |  |  |
| **X12** | 1476 | 2406 | 1917 |  |
| **X13** | 1462 | 2464 | 2009 | 1476 |
