# Supplementary material for: Heritable gene expression differences between apomictic clone members in Taraxacum officinale: Insights into early stages of evolutionary divergence in asexual plants
Source: BMC Genomics. 2016 Mar 8;17:203. doi: 10.1186/s12864-016-2524-6 (PMC4782324; doi:10.1186/s12864-016-2524-6)
Supplement: Additional file 11: — Matrix comparison of Mantel’s tests (r = coefficient of correlation, p = p-value). (DOCX 13 kb) [file 12864_2016_2524_MOESM11_ESM.docx]

**Matrix comparison r p**

Geographic distance – SNP distance 0.183218 0.133333

Geographic distance – expression distance (field, DEG) -0.161220 0.500000

Geographic distance – expression distance (greenhouse, DEG) -0.179580 0.300000

Geographic distance – expression distance (field, ALL genes) 0.118639 0.233333

Geographic distance – expression distance (greenhouse, ALL genes)0.09542 0.300000

SNP distance – expression distance (field, DEG) 0.292980 0.175000

SNP distance – expression distance (greenhouse, DEG) 0.135430 0.316667

SNP distance – expression distance (field, ALL genes) 0.035734 0.350000

SNP distance – expression distance (greenhouse, ALL genes) 0.183935 0.283333
